# Metabolic Barriers to Weight Gain in Patients With Anorexia Nervosa: A Young Adult Case Report

**DOI:** 10.3389/fpsyt.2018.00199

**Published:** 2018-05-18

**Authors:** Verena Haas, Andreas Stengel, Anja Mähler, Gabriele Gerlach, Celine Lehmann, Michael Boschmann, Martina de Zwaan, Stephan Herpertz

**Affiliations:** ^1^Department of Child and Adolescent Psychiatry, Charité — Universitätsmedizin Berlin, Corporate Member of Freie Universität Berlin, Humboldt-Universität zu Berlin, and Berlin Institute of Health, Berlin, Germany; ^2^Department of Psychosomatic Medicine, Charité — Universitätsmedizin Berlin, Berlin, Germany; ^3^Experimental and Clinical Research Center, Charité — Universitätsmedizin Berlin, Berlin, Germany; ^4^Department of Psychosomatic Medicine and Psychotherapy, LWL-Universitätsklinikum, Ruhr-Universität Bochum, Bochum, Germany; ^5^Department of Psychosomatic Medicine and Psychotherapy, Hannover Medical School, Hanover, Germany

**Keywords:** anorexia nervosa, energy requirements, energy metabolism, metabolic chamber, seated non-exercise physical activity

## Abstract

**Background:** Over-proportionally high energy requirements in some patients with anorexia nervosa (AN) have been reported, but their exact origin remains unclear.

**Objective:** To objectively measure metabolic alterations in an AN patient with high energy requirements as judged by clinical observation.

**Materials and Methods:** We present the case of a young woman with AN (index patient, IP; 19 years, admission BMI 13.9 kg/m^2^). After 3 months of treatment at BMI 17.4 kg/m^2^, we assessed her resting energy expenditure (REE), respiratory exchange ratio (RER), diet-induced thermogenesis (DIT), seated non-exercise physical activity (NEPA in Volt by infrared sensors), and exercise activity thermogenesis (EAT) in a metabolic chamber; body composition (bioimpedance analysis), energy intake (15d-food protocol), physical activity (accelerometry) and endocrine parameters. The IP was compared for REE, RER, DIT and seated NEPA to six AN patients (AN-C) and four healthy women (HC-1), and for EAT to another six healthy women (HC-2).

**Results:** Our IP showed high REE (110% of predicted REE according to Harris & Benedict) and high seated NEPA (47% increase over AN-C, 40% over HC-1), whereas DIT (IP: 78 vs. HC-1: 145 ± 51 kJ/180 min) and EAT (IP: 157 vs. HC-2: 235 ± 30 kJ/30 min) were low, when compared with HC. The other AN patients showed a lower REE (AN: 87 ± 2% vs. HC: 97 ± 2% predicted) at increased DIT (AN: 187 ± 91 vs. HC: 145 ± 51 kJ/180 min) when compared with HC. RER of the IP was low (IP: 0.72 vs. 0.77 in AN-C; 0.77 in HC-1 and 0.80 in HC-2).

**Conclusions:** Complex and variable disturbances of energy metabolism might exist in a subgroup of patients with AN during refeeding, which could lead to unexpectedly high energy requirements. Future studies need to confirm the existence, and investigate the characteristics and prevalence of this subgroup.

Clinical trial Registry number: NCT02087280, https://www.clinicaltrials.gov/

## Introduction

Anorexia nervosa (AN) is a severe psychiatric illness, with some patients requiring intensive and repeated inpatient care. The main aim of treatment for patients with AN is to normalize body weight. Recommendations about caloric intakes to gain weight for these patients vary from 30 to 100 kcal/kg/d (1), depending on individual nutritional characteristics, physical activity level and treatment phase.

High caloric requirements for weight gain have been reported in some individuals with AN, i.e., occurring at week 6 of treatment, of >95 kcal/kg/d, which would result in 4,750 kcal for a patient weighing 50 kg (1). However so far, most studies on energy requirements in AN patients rely on the observation of food intake and/or the prediction equations from healthy persons—both of these methods have limited accuracy in patients with AN. Resting energy expenditure (REE) is usually decreased at the start of treatment and increases with weight regain (2). In one individual with AN, REE at the completion of refeeding was reported to be 19% higher than expected for height and weight (3). The exact origin of these high requirements in some AN patients remain unclear.

We hypothesized that in some AN patients, high energy requirements represent a metabolic barrier to weight gain and maintenance. Therefore, we intended to subject an AN patient with high caloric requirements for weight gain, as judged by clinical observation, to extensive and objective metabolic evaluations in order to gain a better understanding of her metabolic status.

## Materials and methods

### Body composition analysis and food intake

Body composition analysis of the index patient was carried out with bioelectrical impedance analysis (BIA-Corpus, Medical Health Care, Karlsruhe, Germany) in combination with the manufacturer electrodes and software (BodyComp V9, scientific-pro). Energy and macronutrient intake of the index patient were assessed over 15 days using food protocols (optidiet, GOE mbH, Linden, Germany).

### Energy and substrate metabolism

**Figure 1:**
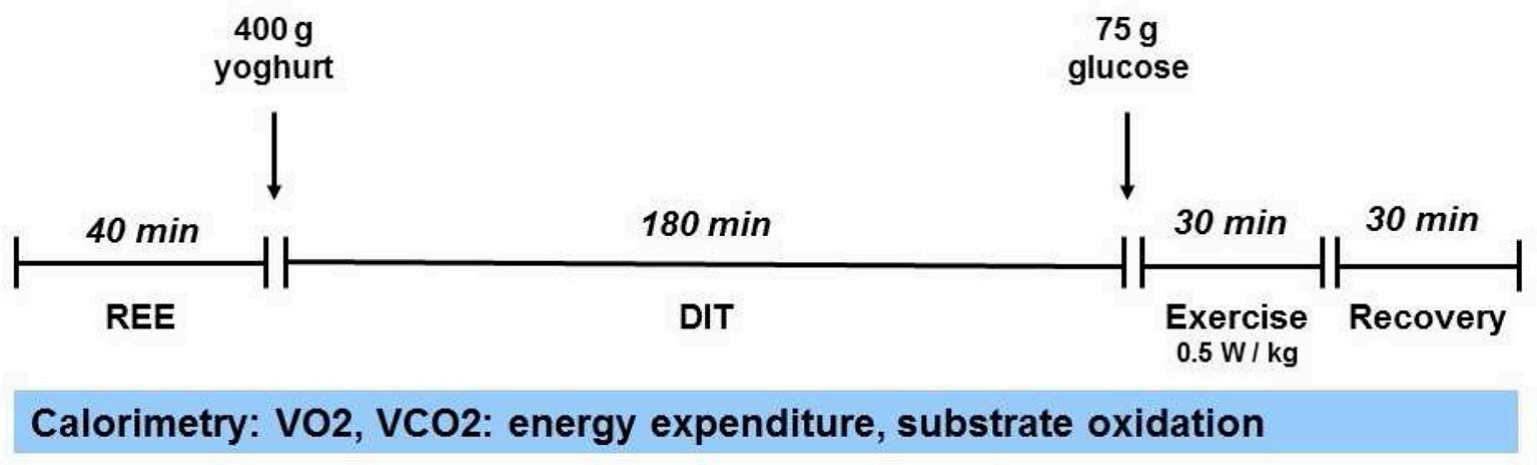
Test protocol in the metabolic chamber. REE, Resting Energy Expenditure; DIT, Diet-induced thermogenesis; VO_2_ and VCO_2_, oxygen and carbon dioxide volumes.

**Figure 2:**
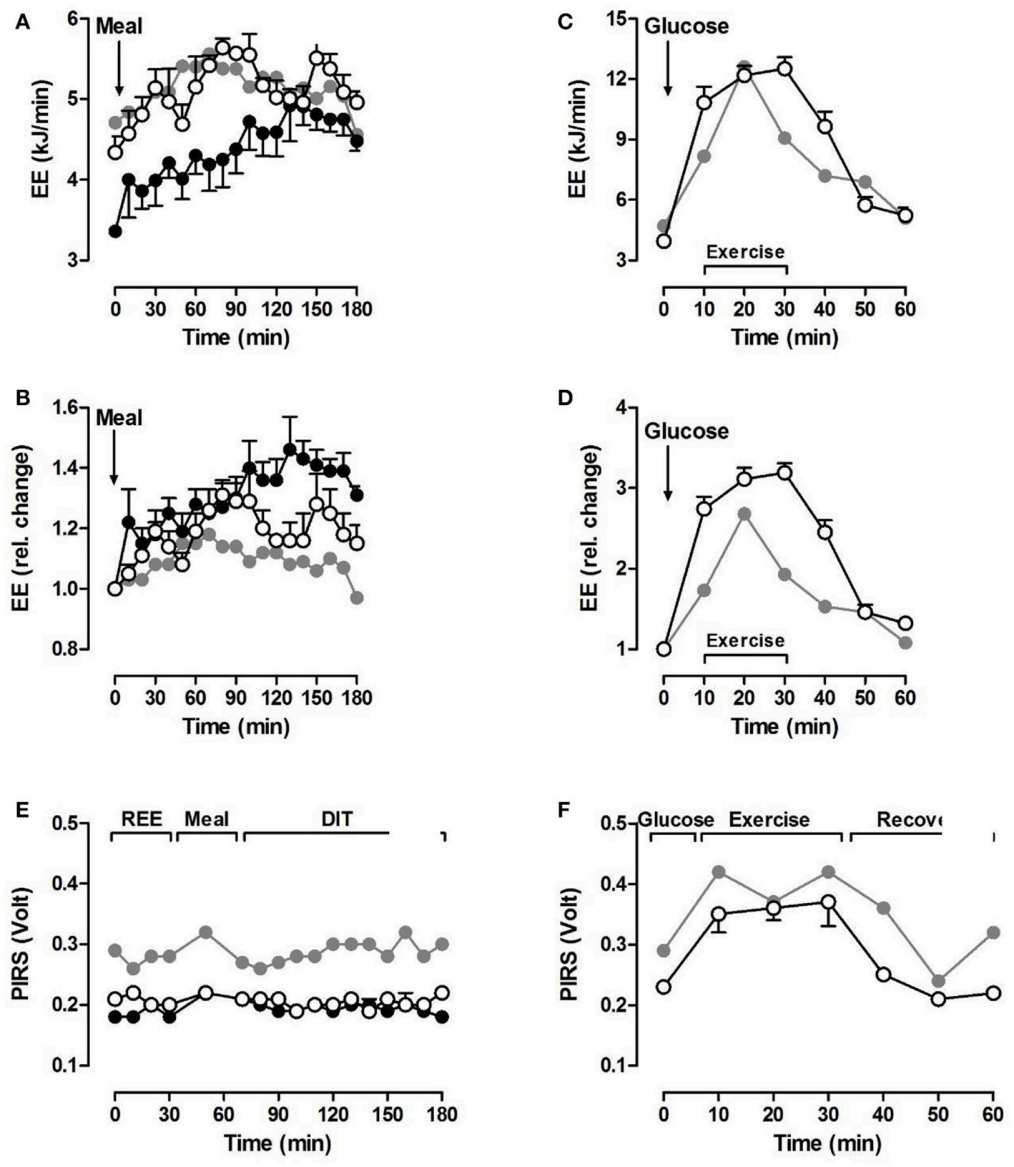
Metabolic test results of the index patient (gray circles) and either 6 AN patients (filled circles) or 4 healthy controls (open circles). Resting (before test meal) and diet-induced energy expenditure (**A**: absolute, **B**: relative change). Exercise activity thermogenesis after a 75 g glucose drink during 30 min on a bicycle ergometer at 0.5 W/kg body weight (**C**: absolute, **D**: relative change).). PIRS: passive infrared motion sensors for assessing non-exercise physical activity inside the metabolic chamber (**E**: while seated; **F**: during exercise). Data expressed as mean ± SEM.

We assessed three major components of energy expenditure (EE) in a metabolic respiratory chamber as described previously (4), i.e., resting energy expenditure (REE) after an overnight 12-h fast, Respiratory Exchange Ratio (RER, ratio between carbon dioxide produced and oxygen consumed (VCO_2_/VO_2_), diet-induced thermogenesis (DIT) after a test meal, and exercise activity-induced thermogenesis (EAT). EAT during 30 min of moderate bicycle exercise is expressed as energy efficiency (workload/EE during exercise) to account for different workloads of the IP and healthy controls. During the entire test protocol, physical activity was recorded by three passive infrared sensors (PIRS) in the chamber. For the detailed test protocol, see Figure 1. Test results of the IP were compared to data from respective control patients from our institutional database: for REE, DIT, RER and seated non-exercise physical activity (NEPA) from six anorexic female patients (AN-C) and four healthy, age-matched women (healthy controls, HC-1); for EAT from another six healthy women (HC-2; Table 1, Figure 2). The AN-C patients had been admitted to inpatient treatment for a mean of 3.7 (range: 2.1–5.4) months and had gained 4.6 (range: 3.2–5.6) kg between admission and the test day (BMI increase from 15.0 to 16.3 kg/m^2^).

**Table 1:** Baseline characteristics and calorimetric measurements of the index patient at rest and after stimulation.

	**Index patient**	**AN-control**	**healthy-control 1 _DIT_**	**Healthy-control 2 _exercise_**
*N*	1	6	4	6
Age (years)	20	20 ± 8	23 ± 1	52 ± 6^**^
Height (m)	1.77	1.62 ± 0.05	1.71 ± 0.03^*^	1.70 ± 0.06^*^
Weight (kg)	54.4	43.0 ± 1.3	62.8 ± 5.8^**^	67.2 ± 3.1^***^
Weight (lbs)	119.7	94.6 ± 2.9	138.2 ± 12.8^**^	147.8 ± 6.8^***^
BMI (kg/mE^2^)	17.4	16.3 ± 0.3	21.4 ± 1.6^*^	23.3 ± 1.6^**^
REE (kcal/d)	1622	1161 ± 68	1492 ± 136^**^	1437 ± 158^**^
REE (% predicted)	109.6	86.9 ± 2.4	97.1 ± 1.7^***^	99.8 ± 8.1^**^
Basal RER	0.72	0.77 ± 0.03	0.80 ± 0.05	0.75 ± 0.04
DIT (kJ/180 min)	78	187 ± 91	145 ± 51	n.d.
Work load (Watt)	30	n.d.	n.d.	40 ± 0
Energy efficiency (%)	34.4	n.d.	n.d.	31.5 ± 4.1
Mean PIRS (Volt)	0.28	0.19 ± 0.01	0.20 ± 0.01	n.a.

*Data of the index patient are compared to 6 AN patients (AN-control) and 4 controls (healthy control 1) after a test meal and to 6 controls (healthy control 2) after 75 g glucose and during 30 min bicycle exercise*.

AN, anorexia nervosa; BMI, Body Mass Index. REE, resting energy expenditure; RER, respiratory exchange ratio; DIT, diet-induced thermogenesis; PIRS, passive infrared motion sensors; EAT, Exercise activity thermogenesis; REE (% predicted according to Harris & Benedict); n.d., not determined; Data are displayed as mean ± SD;

* *p < 0.05*,

** p < 0.01;

*** *p < 0.001 when compared with AN-controls*.

### Physical activity (PA)

PA was assessed by accelerometry using a SenseWear™ armband and software, Version 8.0 (both BodyMedia, Inc., Pittsburgh, PA, USA). PA of the index patient was continuously measured for 5 days in the week after metabolic evaluation.

### Endocrine parameters

We assessed the following hormones of the index patient with a possible modulating effect of PA in AN: cortisol and leptin by the routine laboratory of Charité-Universitätsmedizin Berlin, as well as nucleobindin2 (NUCB2)/nesfatin-1 (# EK-003-26, Phoenix Pharmaceuticals, Inc., Burlingame, CA, USA) and phoenixin (EK-079-01, Phoenix Pharmaceuticals, Inc.) by using ELISA.

### Statistical analysis

To compare metabolic test results of AN-C and HC, the Saphiro-Wilk test was used to test for normal distribution. If data were distributed normally, an independent samples *t*-test was applied, and for non-normally distribute data, the Wilcoxon test.

### Ethics

This study was carried out in accordance with the recommendations of good scientific practice guidelines and approved by the ethical committee of the Charité-Universitätsmedizin Berlin. All subjects gave written informed consent in accordance with the Declaration of Helsinki. In addition, the patient providing the case for the present report gave her written and informed consent for the publication of this case report.

## Results

### Case presentation

A 19-year old woman diagnosed with anorexia nervosa, restrictive subtype (AN; subsequently referred to as index patient, IP) was admitted to the inpatient psychosomatic clinic at the University Hospital Bochum, Germany in April 2015. At that time, her body weight was 44.5 kg (97.9 lbs) corresponding to a body mass index (BMI) of 13.9 kg/m^2^. Amenorrhea was present, a body image disorder was only slightly pronounced. Her estimated premorbid BMI assessed by a patient interview was 23 kg/m^2^ at the age of 15. A previous attempt in a community hospital in order to restore weight had been unsuccessful.

The IP showed oriented awareness, was clear to all dimensions, and appeared in slightly reduced force. Apart from specific eating disorder thoughts revolving around food and weight, formal or substantive thought disorders could be excluded. Alcohol and any drug abuse were denied. At admission, she was on 15 mg mirtazapine per day. Basic serum chemistry including blood sedimentation was within normal limits. Complete blood count indicated a reduced leucocyte number (3.7 × 10^3^/μL). Activities of aspartate (39 U/L) and alanine aminostransferase (29 U/L), and of gamma glutamyl transferase (66 U/L) were within normal ranges. Creatine kinase was elevated (320 U/L). All other laboratory parameters (electrolytes, lipase, amylase, albumin, phosphate, direct and total bilirubin levels, creatinine, glomerular filtration rate, urine analysis) were within normal limits. Thyroid hormones, transglutaminase IgA and endomysium IgA (to exclude celiac disease) were normal. Extensive somatic diagnostics did not indicate any signs for malignancies, gastrointestinal malabsorption, inflammatory bowel disease and endocrine or exocrine pancreatic dysfunction. Clinical chemistry did not show any evidence for a refeeding syndrome at any time.

**Figure 3:**
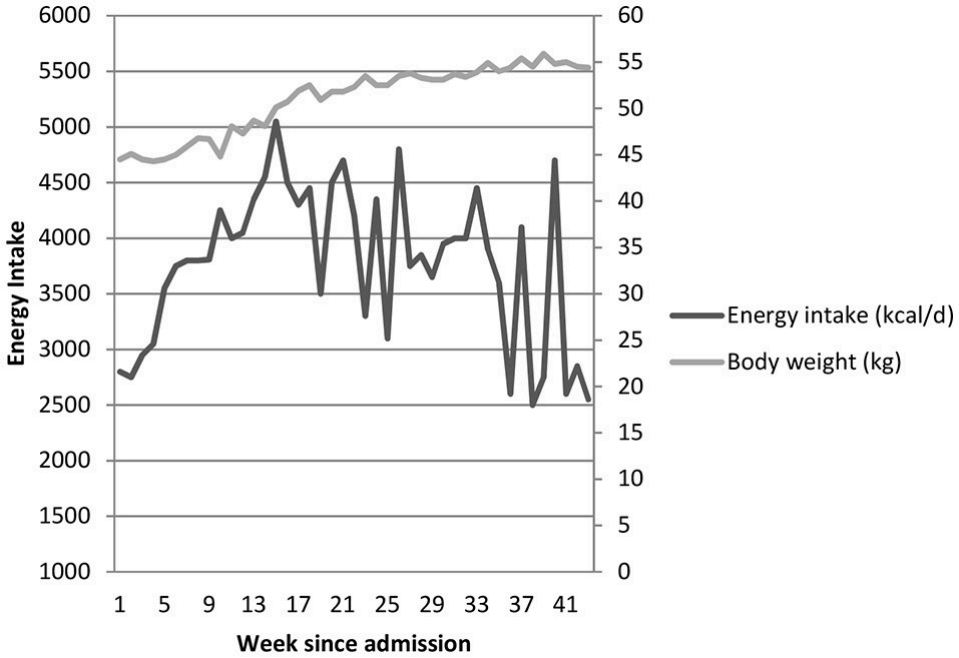
Energy intake (based on information from the patients nutritional diary) and body weight (early morning, fasted, taken weekly) during inpatient treatment.

The IP reported high caloric requirements (4,000 kcal) in order to gain weight. This statement usually raises concerns regarding patients' therapy adherence, because AN patients often avoid weight gain by not eating enough, vomiting, taking laxatives or exercising excessively. The IP denied all counterproductive behaviors. Thus, after initial low energy intake, her meal plan in the later refeeding phase was adjusted to 4,000 kcal/d resulting in a steady increase in body weight (Figure 3). Usually at this stage, our patients achieve weight gain with considerably lower caloric intakes (around 2,500–3,000 kcal/d).

### Metabolic evaluation

In July 2015, the IP was referred to the metabolic ward at the Charité-Universitätsmedizin Berlin in order to test for any disturbances within the regulation of energy metabolism. Body composition analysis indicated a fat mass of 8.6 kg (18.9 lbs, 15.8% of body weight), which is about 13% lower than in normal weight healthy young women. Fat free mass was 45.8 kg (100.8 lbs) and hence within the low normal range. Mean energy intake assessed over 15 days by food protocols was 3,940 kcal/d. This was achieved by eating six meals daily, including a late evening meal consisting mostly of sweets (i.e., 325 g of cookies and four chocolate bars) or full fat sweetened dairy products (i.e., 500 g of ice cream and 200 g of chocolate). Physical activity as assessed with accelerometry yielded an estimated 24-h EE of our IP of 2,632 kcal/d with 14.967 steps per day.

#### Resting energy expenditure (REE)

Surprisingly, REE of our IP was increased compared to both AN-C and HC-1 (Table 1). RER values close to 0.70 indicate almost 100% fat oxidation, a value close to 1.00 almost 100% carbohydrate oxidation. Interestingly, with 0.72 basal RER of the IP was low (Table 1).

#### Diet-induced thermogenesis (DIT)

The increase in EE in absolute values of the IP after the test meal seemed comparable to HC-1, at least within the first 120 min after the meal (Figure 2, left, top). However, the relative increase above REE indicated that there was just a 1.2-fold increase within the first 60 min after the meal followed by a decrease reaching baseline values within the next 120 min. In HC-1, EE increased about 1.3-fold within 90 min and in AN-C about 1.4-fold with 120 min after the meal (Figure 2, left, middle). Therefore, DIT was highest in AN-C and lowest in the IP.

#### Seated non-exercise physical activity (NEPA)

According to the PIRS data, the IP showed a much higher seated NEPA level compared to AN-C and HC-1 (Figure 2, left, bottom).

#### Exercise activity thermogenesis (EAT)

During low intensity bicycle exercise after a glucose load, EE increased about 3-fold within the first 10 min in HC-2 and remained at that level until the end of testing (Figure 2, right, top and middle). In the IP, EE also increased within the first 20 min of exercise followed by a rapid decrease within the next 10 min. PIRS signals during exercise were comparable between the IP and HC-2 (Figure 2, right, bottom).

### Endocrine data

In the week of metabolic testing, cortisol of the IP was high (452 nmol/l vs. reference range of 124–388 nmol/l). Phoenixin of the IP was 0.31 ng/ml (reference range unknown), while leptin was in the low normal range (2.2 vs. reference of 1.0–5.6 μg/l). With 0.64 ng/ml NUCB2/nesfatin-1 was high (reference range ~ 0.2 ng/ml). In addition, thyroid hormones were assessed 2 weeks prior to metabolic testing and yielded the following results: FT3, 2.3 pg/ml (reference of 2.0–4.4); FT4, 1.1 ng/dl (reference of 0.9–1.7); TSH 2.21 (reference of 0.27–4.20).

### Further course of treatment

Body weight course during inpatient treatment as well as caloric intake of the IP (data derived from her personal nutritional diary) are shown in Figure 3. Her intake ranged between 46 and 101 kcal per kg body weight, was never below 2,500 kcal/d, with a peak intake of 5,000 kcal/d. After the tests, the IP was continued on a high energy meal plan and discharged in September 2015 with a body weight of 54.7 kg (120.3 lbs, BMI 17.5 kg/m^2^). We recommended specific nutrition counseling after discharge to meet increased energy requirements. A brief email-interview with the IP was carried out 14 months after metabolic investigations to gain her prespective on the tests (interview is provided as appendix).

## Discussion

This case report indicates that there might be complex and largely variable disturbances of energy metabolism in some AN patients during refeeding. The index patient (IP) showed increased resting energy expenditure (REE)b and seated non-exercise physical activity (NEPA), whereas diet- and exercise-induced thermogenesis were decreased. In contrast, the other AN patients showed a decreased REE, normal seated NEPA and increased diet-induced thermogenesis. With 2,500–5,000 kcal/d (46–101 kcal/kg/d), the energy intake during inpatient treatment reported by the IP was high. This high requirement for weight gain in some individuals with AN is in accordance with earlier studies (1, 5). The strength of our study is the direct and objective assessment of energy metabolism explaining the high requirements of the index patient (IP).

Hypermetabolism can be defined as an REE above 110% of predicted values according to weight, height, age and gender (6). In acute AN, REE is usually decreased due to metabolic adaptation to starvation, i.e., to 70 ± 18 (50–93)% of predicted REE and increases to 102 ± 13 (94–119)% at completion of refeeding (3). Interestingly, despite sitting obviously relaxed in an armchair, our IP showed high non-exercise physical activity (NEPA) when measuring REE and DIT as indicated by the three PIRS suggesting increased seated NEPA by almost a factor of 1.5 compared to healthy controls and other AN patients. A recent study using a novel shoe-based monitor to detect “fidgeting” (seated NEPA) in AN patients also reported increased activity values for AN patients at different stages of inpatient treatment when compared with matched controls (7). Possibly, in the situation of increased seated NEPA, REE might have been overestimated in our study. While increased NEPA, induced by walking, standing and fidgeting is commonly observed in AN patients treated in clinical settings and could explain why some patients are resistant to weight gain, very few studies exist that specifically and objectively assessed and quantified this form of PA in AN patients

DIT of our IP was substantially reduced and no more measurable at the end of testing. In both control groups, DIT was still active at the end of testing. In another study, DIT was rather increased in AN patients during early refeeding (8). Reasons for a reduced DIT in our IP (tested in the late refeeding phase) might be impairments in nutrient digestion, resorption, transport and storage. Possibly, a change in gut microbiota might affect energy needs, as maintenance of gut bacterial population requires substantial amount of energy (9). Taking into account the increased seated NEPA, DIT might have been even lower in the IP. Other authors hypothesized about a “metabolic waste phenomenon” in AN, such as low efficiency of lean tissue for energy storage (8), and a decreased hepatic glucose oxidation capacity during weight restoration (10). With 0.72 basal RER of the IP was low when compared to both control groups. A high RER indicates low fat oxidation capacity and has been linked to higher rates of weight gain in AN (10) and obese subjects (11).

We compared the physical activity of the IP with 39 adult AN inpatients assessed in our unit before (12), showing moderate (6331 ± 423 steps/d), high (13,743 ± 1047 steps/d), and sometimes excessive PA (up to about 26,000 steps/d). Thus with 14,967 steps/d, physical activity of our IP was high, but not excessive. However, EAT of our IP during testing in our metabolic chamber was lower when compared to healthy controls. Possibly, the preceding glucose load was not sufficient for fueling EAT and internal energy stores could not sufficiently be mobilized.

In past studies, increased PA in AN was associated with hypercortisolemia (13), while for leptin conflicting data exist (14, 15). In the IP, cortisol was slightly elevated and leptin in the low normal range. Phoenixin and nesfatin-1 both are recently discovered peptides associated with an effect on locomotor activity in rats (16, 17). Whether these experimental findings can be translated to humans requires further investigation. So far no reference values exist in humans for phoenixin. NUCB2/nesfatin-1 levels of our IP were 0.64 ng/ml, which is considerably higher compared to previous studies (mean ± SD: 0.22 ± 0.19 ng/ml) in AN patients (18) and may contribute to the increased energy expenditure observed here as suggested in animals before (19).

### Clinical implications and future directions

Inpatient AN therapy has limitations, about 30–50% of patients require re-admission within 12 months after discharge due to renewed weight loss (20). Psychological variables such as persisting body dissatisfaction, but also biological mechanisms might contribute to the difficulties to gain and maintain weight. Earlier studies suggested abnormally high caloric requirements in some patients with AN (1, 21, 22). However, these studies relied on observing food intake and predicting caloric intake—both techniques with limited validity.

In obesity decreased REE after dieting accounts for quick weight regain (“yoyo-effect”). Homeostatic responses to weight perturbation encompass coordinated changes in behavior and vegetative systems controlling EE. These responses are coordinated in a manner to oppose weight perturbation in either direction (23). Concomitantly, experimental overfeeding in healthy male volunteers increased EE, using up to 70% of the extra energy ingested. Two-thirds of the increases in total daily EE was due to increased non-exercise activity thermogenesis (NEAT), and changes in NEAT accounted for the 10-fold differences in fat storage that occurred and directly predicted resistance to fat gain with overfeeding (24). The longer an experimental intervention of experimental overfeeding in healthy men, the higher was the difference between expected and actual weight gain (25). Resistance to continuous weight gain and maintenance, particularly in the late treatment phase, would have crucial therapeutic implications for AN patients. Over 20 years ago, Vaisman et al. pointed out that a better understanding of energy metabolism during refeeding AN patients is necessary to provide a rational basis for nutritional management (26). However, so far metabolic considerations are not incorporated in AN therapy (1). We hope that the present study will raise awareness within a broad forum of experts and care providers working with AN patients, that a lack of weight gain in AN could—at least in a subgroup of patients—be of biological origin. Not correctly identifying or accusing these patients of manipulation will strain their relation to the therapeutic team, decrease therapy adherence and success and ultimately promote relapse.

It is our intention to strengthen metabolic research in the eating disorder field, by pointing out the following future directions:

1. To confirm the existence of the above mentioned biological barriers to weight gain in AN patients by investigating larger AN populations at different and well-defined phases of nutritional recovery, and to assess their effects on the therapeutic outcome
2. To better characterize the biological origin of high energy requirements in some AN patients, by identifying
  a) biological phenotypes with high energy requirements before the onset of AN or at the start of therapy
  b) the effect of different components of energy expenditure including non-exercise physical activity
  c) the effect of relative “overfeeding” or “over-consumption” of energy dense food during treatment
  d) alterations in gut microbiota
3. To use objective measurement techniques to quantify EE in AN patients rather than to rely on observations of energy intake and to carefully select comparable patients and treatment phases

### Study limitations

Our findings are based on a single case and need confirmation in further studies. In addition, we were not able to provide data on the metabolic status of the IP during weight loss or at the start of treatment. There were two methodological limitations: first, the control group for EAT was not age matched, which could have an influence when comparing the results. Second, we assessed body composition only in the IP, and high fat free mass could be at least one reason for her high REE. However, we believe that the movement registration by infrared sensors and the resulting high SPA was unaffected by body composition.

## Author contributions

Study conception: VH, SH, MdZ; Study design: VH, MB, AM, AS; Data acquisition: AM, GG, VH, AS, SH, CL; Data analysis and interpretation: AM, MB, VH, AS, CL; Writing the paper draft: VH, MB; Critical revision of the manuscript: SH, MdZ, AS.

### Conflict of interest statement

The authors declare that the research was conducted in the absence of any commercial or financial relationships that could be construed as a potential conflict of interest.

## Appendix

**Patient perspective** (14 months after metabolic assessment, via email)

**Question 1**: How would you evaluate your participation in the study in retrospect?

**Answer 1**: I thought it was fun to be part of a research project. Moreover, the study made it clear to me, that general recommendations about energy needs that you can find in the internet are not reliable. Before the study, I used these recommendations as orientation, and first, I found it hard to believe, that my energy expenditure could be so much higher. Yet, the study results showed just that. On one hand, the high energy expenditure was a burden for me during my time in the clinic. I often had to eat despite not being hungry or having appetite. Because I consumed most calories in the evening as sweets, I often went to bed feeling very sick, unwell and with hot flashes. It also made it difficult for me to fulfill the treatment contract in terms of the expected weekly weight gain. On the other hand, it was good to know that I could and had to eat a lot to gain weight. One part of me also enjoyed being able to eat so much.

**Question 2**: Where do you see room for improvement of nutritional therapy for AN patients?

**Answer 2**: I think it would be important that patients with high energy expenditure like me should be recognized earlier and be taken more seriously by professionals. I am well aware that many AN patients often just claim that they eat a lot and still do not gain weight, or that this is their subjective perception. Accordingly, these statements cause skepticism in the treating teams. For me, it was helpful to get two special permits during my inpatient stay: first, the expected rate of weight gain was reduced by 200 g. Second, I was allowed to give sweets, dry fruit and nuts into storage with the staff, which I could pick up whenever I wanted to. For me it was not an option to cover the extra calories by liquid meals. Actually, I like eating, therefore I wanted to be able to gain my weight by eating enjoyable food, that before I had not permitted myself to eat for months.
